# Backpacks & Briefcases, Walkers & Wheelchairs? University Expansion & Housing Justice for Urban Older Renters

**DOI:** 10.1093/geroni/igaf122.440

**Published:** 2025-12-31

**Authors:** Ian Johnson

**Affiliations:** The University of Texas at San Antonio, San Antonio, Texas, United States

## Abstract

University expansion and district revitalization often exacerbate existing housing inequities, particularly for urban-dwelling older adult renters. This presentation explores the university’s role in housing justice, focusing on the impacts of institutional growth on vulnerable older populations. This presentation will present a media analysis of university-driven development in downtown San Antonio and how it has prompted and responded to concerns regarding displacement, rent escalation, and diminished housing security among older residents. Findings reveal that university expansion efforts, while intended to stimulate economic growth, frequently overlook the specific needs of long-term older renters. Further, language and rhetoric used to frame district planning similarly excludes older adults as stakeholders in urban places. We will discuss the complex interplay between university planning, local policy, and the lived experiences of older adults, highlighting the challenges of maintaining age-friendly communities amidst rapid urban change. This presentation aims to present a framework for actionable strategies for universities and policymakers to adopt ethical and equitable approaches to urban redevelopment. We will offer considerations for integrating age-friendly principles into university district planning, emphasizing the importance of community engagement, affordable housing preservation, and the protection of older adults’ rights to remain in their neighborhoods.

